# TiO_2_ Nanotubes: Recent Advances in Synthesis and Gas Sensing Properties

**DOI:** 10.3390/s131114813

**Published:** 2013-10-31

**Authors:** Vardan Galstyan, Elisabetta Comini, Guido Faglia, Giorgio Sberveglieri

**Affiliations:** Sensor Lab, Department of Information Engineering, University of Brescia and CNR INO, Via Valotti 9, 25133 Brescia, Italy; E-Mails: guido.faglia@ing.unibs.it (G.F.); giorgio.sberveglieri@ing.unibs.it (G.S.)

**Keywords:** titanium dioxide, nanotubes, electrochemical anodization, gas sensors

## Abstract

Synthesis—particularly by electrochemical anodization-, growth mechanism and chemical sensing properties of pure, doped and mixed titania tubular arrays are reviewed. The first part deals on how anodization parameters affect the size, shape and morphology of titania nanotubes. In the second part fabrication of sensing devices based on titania nanotubes is presented, together with their most notable gas sensing performances. Doping largely improves conductivity and enhances gas sensing performances of TiO_2_ nanotubes.

## 1. Introduction

The development of portable and low cost gas sensors with high sensitivity, selectivity, and low working temperature is highly desirable and still a great challenge. Metal oxides are attractive materials for the fabrication of gas sensing devices because of their obvious advantages, such as low cost, production flexibility, and good thermal and chemical stability [1,2]. Especially hierarchical nanostructured metal oxides with the different morphologies are good candidates for the manufacture of gas sensors.

The conductivity of metal oxide nanostructures changes with the surface adsorption and desorption of gas molecules. This change is caused by the electronic transfer that occurs upon the adsorption of gas molecules over the film surface [3,4]. These conductivity changes strongly depend on the shape and the size of the nanostructures. Recently TiO_2_ nanostructures with the different shapes have received extensive attention from the gas sensing research community due to their unique physical and chemical properties [5–9], and nanostructured titania with tubular shape has been considered one of the most promising materials for the fabrication of gas sensing devices [10].

The gas sensitivity can be enhanced by the large surface area and the morphology of tubular structures. For reproducibility of the functional properties of gas sensors the preparation of highly ordered nanostructures is also an important factor. Investigations have shown that titania nanotubes are sensitive mainly towards hydrogen [11,12]. To expand the applications of titania nanostructures in the fabrication of chemical sensors there are number of parameters that need to be improved, namely the conductance of TiO_2_ in air, the sensing signal, the response and the recovery times.

Extensive efforts have been made to extend the functionalities of titania by modifying the band structure with dopant materials [13–19]. Investigations show that doped and mixed structures of titania are emerging as important materials for the improvement of conductometric sensors' properties [20–27]. The change of carrier density by different dopants in polycrystalline TiO_2_ has been investigated. A typical dopant is Nb, which acts as a shallow donor in TiO_2_ [28,29]. Compared to other dopants such as Pt, carbon nanotubes, Pd, P and Ni [18,20,21,26], the similarity of the radii of Nb^+5^ (r = 0.70 Å) and Ti^+4^ (r = 0.68 Å) allows Ti atoms to be replaced by Nb in the lattice [30]. Chemical sensors operate on the basis of the adsorption and removal of oxygen on the surface of the material. To enhance this adsorption/desorption effect on the TiO_2_ surface the working temperature of the sensing layer must be higher than the normal ambient air temperature, but the phase transformation from anatase to rutile at high temperature ranges can cause a drastic decrease in sensor properties [31], as it has been found that the anatase phase of titania is more reactive than the rutile one [32–34]. The experimental results have shown that the presence of substituted Nb ions in the anatase structure of TiO_2_ inhibits the undesirable phase transition of the structure [30], so to enhance the conductivity and to stabilize the gas sensing properties of titania Nb is more preferable compared to other dopants.

The development of fabrication technics for the synthesis of highly ordered doped or mixed titania nanostructures is very current. Since the formation of titanium dioxide nanotubes by electrochemical deposition in a mould was reported in 1996 [35], numerous investigations have been carried out to develop fabrication methods for titania tubular structures. The present review is focused on the recent developments in the synthesis, modifications and gas sensing properties of titania nanotubes which can open new perspectives for the fabrication of gas sensors.

## 2. Synthesis of Titania Nanotubes

A few approaches have been employed for synthesis of titania tubular structures such as electrochemical anodization [36–39], atomic layer deposition (ALD) [40–42] and hydrothermal synthesis [43–45]. Detailed description of the preparation methods is given in Table 1. For the preparation of TiO_2_ nanotubes by means of ADL it is necessary to use a porous template. High quality nanoporous anodic aluminum oxide is usually used as template to promote the growth of titania nanotubes by ADL. The template-assisted technique may limit the choice of the substrate and requires post-processing separation of the obtained tubular arrays from the template. In addition part of the tubes may break off from the substrate during the template removal [40]. Hydrothermal synthesis is a method consisting of hydrothermal treatment of solutions of anatase and rutile titania powders and post-growth annealing. Preparation of titania nanotubes by means of electrochemical anodization refers to the anodic formation of titania nanotubes by oxidation and etching of metallic titanium. The method, described below in detail, allows direct growth of the TiO_2_ tubular arrays on different types of substrates and the modification of the surface structure of titania at room temperature [39,46].

### 2.1. Anodic Formation of Titania Nanotubes

**Figure 1.**
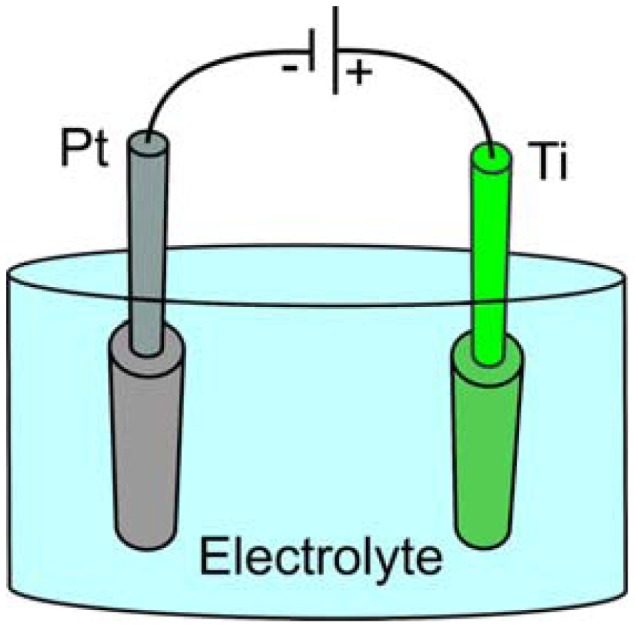
Schematic drawing of the electrochemical cell for the anodization. It is two-electrode system where the platinum is a counter electrode and the titanium is the anode. The tubular arrays of TiO_2_ are formed by the oxidation and etching of the metallic titanium in the electrolyte. Anodization is carried under the voltage applied between the anode and the counter electrode.

Electrochemical anodization of titanium is a relatively simple and efficient process to fabricate well-aligned and highly ordered TiO_2_ tubular structures. Anodization is carried out in a two-electrode system and the process is controlled by variation of the anodization parameters (Figure 1).

**Figure 2.**
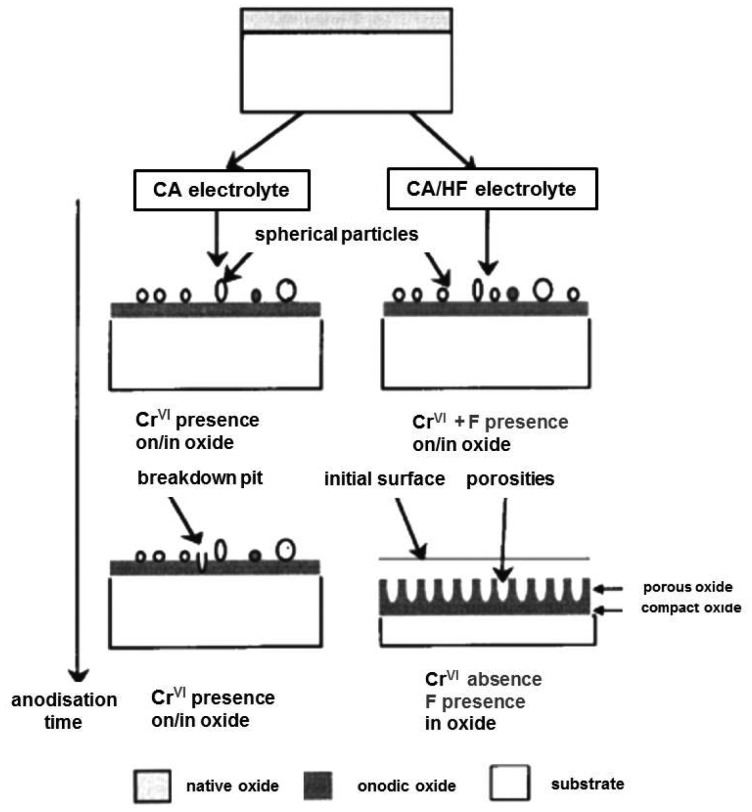
Proposition of a growth mechanism. Reprinted from [49] with permission. Copyright (2013) John Wiley … Sons, Inc.

Preparation of titania tubular arrays by means of the anodization method in chromic acid solution both without and with hydrofluoric (HF) acid addition was performed for the first time by Zwilling *et al.* in 1997 [48,49]. The potential between the specimen and a titanium cathode was increased from 0 to 5 or 10 V in five equal steps of 1 min each. Zero volts means that the anode and the cathode were short-circuited. Then the specimen was maintained at the final voltage for variable times ranging between 1 and 55 min; some experiments were performed by reaching the final voltage in a single step. Non-porous oxide compact films were formed after the anodization of titanium and titanium alloy in chromic acid without HF and a duplex film composed of a compact layer surmounted by a columnar porous layer have been formed in chromic acid containing HF. When fluorine was not present the Cr(VI) species played a poisoning role and stopped the growth of the compact non-porous layer formed in chromic acid media (Figure 2) [49].

In HF-containing solution the fluorine ions played an antidote role and the local competition between Cr(VI) and F^−^ species led to a continuous growth of the porous structure (Figure 2). The consistency of the oxygen content was explained by a compensation between film thickening and pore extension due to the competition between dissolution and growth [49]. Thus, the oxidation and the dissolution of the metal are the key processes for the formation of titania nanotubes by electrochemical anodization. The anodization process can be described as follows: in the first step an oxide barrier layer is formed on the electrolyte-metal interface [39,50]:

(1) $$Ti^{4+}+2H_{2}O\to TiO_{2}+4H^{+}$$

Then cracks and narrow slits appear on the surface due to field-enhanced dissolution of the oxide layer. Diffusion of the electrolyte into these cracks and slits enhances the dissolution rate compared with other areas:

(2) $$TiO_{2}+6F^{-}+4H^{+}\to {\left[TiF_{6}\right]}^{2-}+2H_{2}O$$

**Figure 3.**
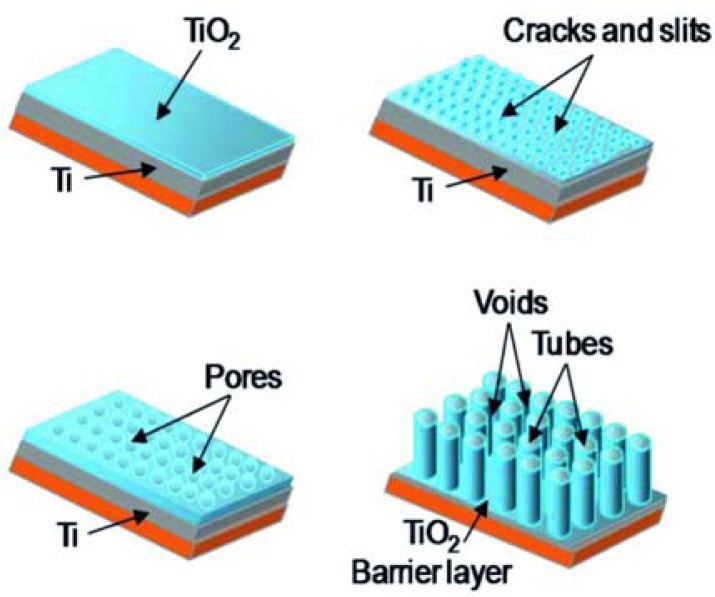
Schematic diagram of the evolution of TiO_2_ nanotube arrays by means of potentiostatic mode anodization. Reproduced from [39] by permission of The Royal Society of Chemistry.

The growth of nanotubes is related to the diffusion of F^−^ ions through the oxide layer and effusion of [TiF_6_] ^2−^. Therefore by variation of the content of water and fluorine ions in the electrolyte it is possible to control the titanium anodization process [39,50]. Cracks enlarge and become connected with neighboring cracks. Initial pores are formed in the cracks. Then a random formation of a porous structure in the cracks and slits occurs (Figure 3). As the anodization proceeds, the interpore regions are also attacked by the F^−^ ions. Therefore slits are generated at those parts. The growth of those slits leads to the formation of parallel tube-like arrays [39].

Morphological and structural studies show that the rates of the reactions (1) and (2) are strongly affected by the type and the pH of the electrolyte, the applied voltage (or current) and the anodization temperature. In reaction (1) the Gibbs free energy ΔG_el_ change obeys the Nernst equation and is a function of the electrode potential; ΔG_el_ = −zFE (3) where z is the electron transfer number, F is the Faraday constant, and E is the electrode potential, which is changed by the applied voltage [51]. In the electrochemical cell most of the applied voltage is consumed in the current–resistance drop of the conducting medium and a relatively small fraction of it manifests as the polarization potential on the electrodes. The absolute value of the over-potential on electrode is expected to increase with increasing applied voltage. The chemical dissolution of TiO_2_ occurs via reaction (2). Fluoride ion is a potent species that can break down metal oxides, *i.e.*, cause metal oxide dissolution reactions when Gibbs free energy change is negative (ΔG^0^_ch_). ΔG_ch_ is Gibbs free energy change of chemical dissolution of the metal oxide. When ΔG_ch_ < ΔG_el_ < 0, the metal oxide formed is protected by the electrical potential against chemical dissolution. In the proximity of the cross-point between ΔG_el_ and ΔG_ch_, the anodized metal oxide is in part dissolved and in part protected, leading to the formation of a porous oxide [51], hence the geometrical parameters of the resulting titania tubular structures are strongly affected by the anodization conditions.

Further the formation of porous TiO_2_ on titanium was investigated in aqueous solutions of HF and H_2_SO_4_/HF electrolyte [36,52]. In aqueous solution of HF the titanium oxide nanotube arrays were obtained under anodizing voltages ranging from 10 to 40 V. In all cases, the final length of the nanotubes was not affected by the anodizing time [36]. Beranek *et al.* also prepared highly ordered porous TiO_2_ with single pore diameters of 140 nm in H_2_SO_4_/HF electrolyte. During the formation process, significant current oscillations were observed with an amplitude which depended strongly on the HF content of the electrolyte. Investigations had shown that due to the high rate of chemical dissolution of TiO_2_ in this solution, the nanotubes grown up to a limiting thickness of ∼500 nm [37].

**Figure 4.**
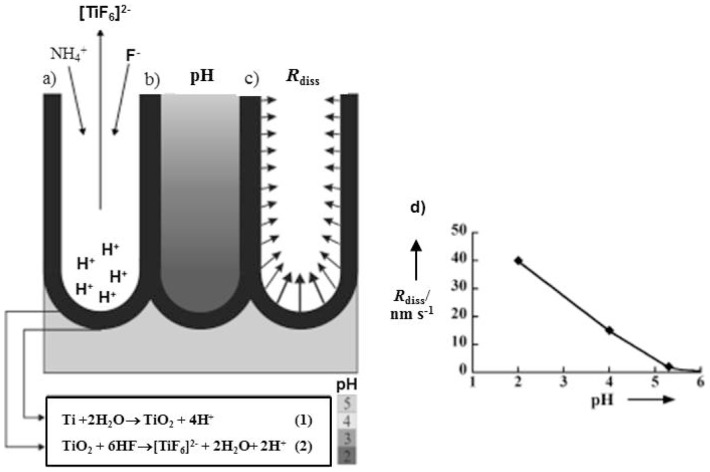
Schematic representation of the dissolution reactions and mechanisms (**a**), the pH profile within a pore (**b**), the dissolution-rate profile within a pore wall (**c**). Experimental determination of the dissolution rate, R_diss_, of the anodic TiO_2_ depending on the pH value (**d**), results are taken from XPS sputter profiles of 20 V anodic oxide immersed for different times in 1m (NH_4_)_2_SO_4_ + 0.5 wt% NH_4_F solution with different pH values. Reprinted from [52] with permission. Copyright (2013) John Wiley & Sons, Inc.

Afterwards Macak *et al.* reported that the chemical dissolution rate of TiO_2_ is highly dependent on the pH value of the electrolyte [52]. They demonstrated that the thickness of the porous layer is essentially the result of equilibrium between the electrochemical formation of TiO_2_ at the pore bottom and the chemical dissolution of this TiO_2_ in an F^−^ ion containing solution (Figure 4). Paulose *et al.* confirmed that the nanotube length is a function of both pH and the anodization voltage. By increasing voltage at a given pH value the tube length and the pore size were increased (Table 2) [8].

Varghese *et al.* increased the thickness of tubular layer by anodization of titanium in fluorine-containing dimethyl sulphoxide (DMSO) electrolyte [53]. The length of tubes was between 0.3 and 33.0 μm. During the experiments ethylene glycol (EG) was also used as an electrolyte besides the fluorine-containing DMSO. They found that the conductivity of an electrolyte composed of hydrofluoric acid (HF) and DMSO is originally low, but can be enhanced substantially by applying an electric field between two immersed electrodes and the oxide dissolution rate during anodization of titanium films in this electrolyte is proportional to its conductivity. To obtain longer nanotubes, the electrolyte conductivity should be lower. DMSO was found to be optimal for obtaining nanotubes of high structural and optical quality with a wide range of lengths compared to EG-based electrolytes.

**Figure 5.**
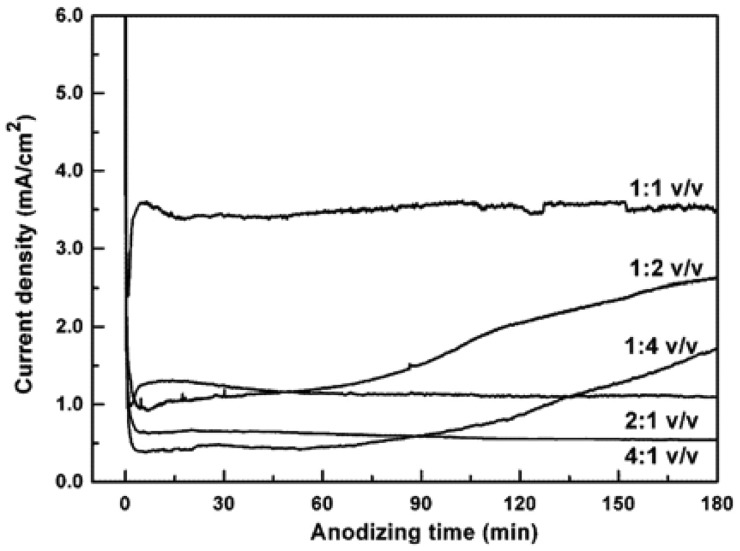
Current density-anodizing time curve for electrochemical anodization in the mixed solution containing glycerol and deionized water at different volumetric ratios at 10 V for 3 h. Reprinted from [38] with permission. Copyright (2013) Elsevier B.V.

Lai *et al.* investigated the growth rate of TiO_2_ nanotube arrays in novel organic–inorganic electrolyte system [38]. The electrolytes were 0.5 wt% NaF and 0.2 M Na_2_SO_4_ in a mixed solution containing glycerol (1,2,3-propanetriol) and deionized (DI) water. Dimensions and morphology of the anodized TiO_2_ were strongly dependent, on the volumetric ratios of glycerol *versus* water, besides on the applied potential and the anodizing time. The current density-anodizing curve was recorded during the anodization of Ti foil in the water–glycerol electrolyte for different volumetric ratios at 10 V (Figure 5). The values of current densities were significantly different, thus the growth rates of TiO_2_ nanotube arrays were strongly different.

**Figure 6.**
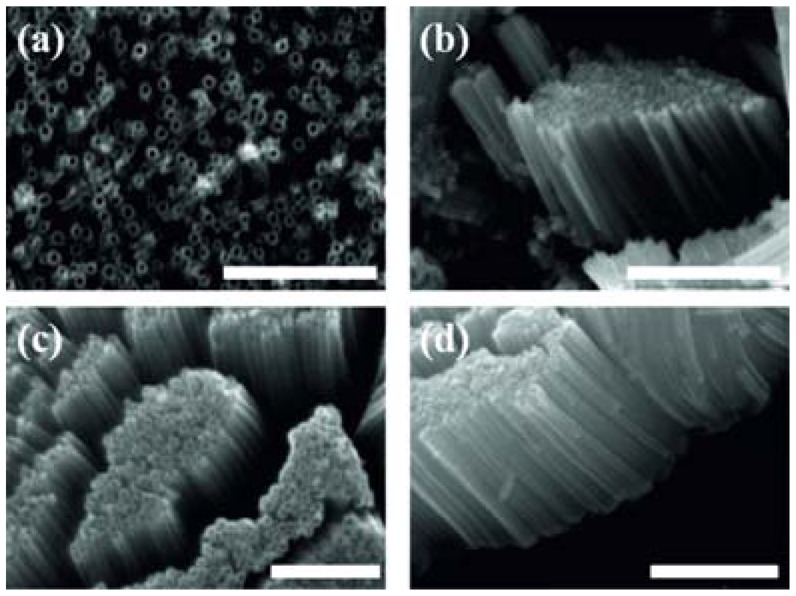
SEM images of TiO_2_ nanotube arrays obtained by anodizing Ti foil in glycerol electrolyte at 40 V at (**a**,**b**) room temperature, scale bar = 2 μm for (a) and 5 μm for (b); (**c**,**d**) in an ice bath, scale bar = 500 nm for (c) and 1 μm for (d). Reprinted with permission from [54]. Copyright (2013) American Chemical Society.

Wang *et al.* reported that in non-aqueous electrolyte the TiO_2_ nanotube dimensions are affected by the electrolyte temperature [54]. They anodized titanium foil in aqueous (*i.e.*, 0.5 wt% hydrofluoric acid in DI water) and nonaqueous electrolytes (*i.e.*, glycerol containing ammonium fluoride); Anodization was performed at room temperature and inside an ice bath. According to the obtained results in nonaqueous electrolyte (*i.e.*, glycerol), the nanotube diameter is markedly affected by the electrolyte temperature (Figure 6). At lower temperature (*i.e.*, in the ice bath), the fluorine ion mobility in the viscous glycerol electrolyte was further suppressed, resulting in much slower etching of formed TiO_2_, which in turn led to a smaller nanotube diameter.

Recently Galstyan *et al.* have used different kind of electrolyte to investigate the effect of ion mobility on the anodization process [39]. H_2_SO_4_ aqueous solution (0.5–1 M) and H_2_O in glycerol (1–5 M) with NH_4_F (0.5–1 wt%) have been used as electrolytes. Nanotubular and nanoporous TiO_2_ structures have been prepared on different substrates by anodization of Ti films. For the fabrication of the tubular and the porous structures the anodization has been carried out in constant voltage (potentiostatic) mode and constant current (galvanostatic) mode at 20 °C.

Well-aligned TiO_2_ nanotubes have been obtained in glycerol-based electrolyte by potentiostatic mode on a variety of different substrates: flexible polyethylene terephthalate (PET), smooth and rough alumina, and titanium sheet. Nanoporous structures have been obtained in H_2_SO_4_ aqueous solution by potentiostatic mode.

Porosity and pore diameter were strongly dependent on current density and anodization time. In H_2_SO_4_-containing aqueous electrolyte a variation in pore size up to ∼80 nm has been obtained, while in NH_4_F-containing glycerol electrolyte the variation has been no more than 30 nm. The anodization process is slow for NH_4_F-containing glycerol electrolyte due to the high dielectric constant and coefficient of viscosity, hence the dependence of the diffusion constant on the viscosity follows the Stokes–Einstein equation: D = k_B_T/(6πηr_s_), where D is the translational diffusion coefficient, k_B_ is Boltzmann's constant, T is the absolute temperature, η is the dynamic viscosity, and r_s_ is the radius of a spherical body. According to this formula, D is inversely proportional to η, therefore the differences between pore diameters for the samples anodized in NH_4_F-containing glycerol electrolyte has been less than in H_2_SO_4_ aqueous electrolyte, due to the different viscosity of the two solutions.

The influence of the electrolyte viscosity on the growth rate of TiO_2_ nanotubes was also investigated by Sreekantan *et al.* [50]. Anodization was carried out in ethylene glycol- (η = 16 cP at 25 °C) and glycerol (η = 945 cP at 25 °C)-based electrolytes. The growth rate of the nanotubes was higher in ethylene glycol because of it's low viscosity. The length of nanotubes was increased when 1 wt% water was added to the ethylene glycol, but when water content was increased to 2 wt% the length of tubes decreased. The calculated rate of formation was approximately 308 nm min^−1^.

**Figure 7.**
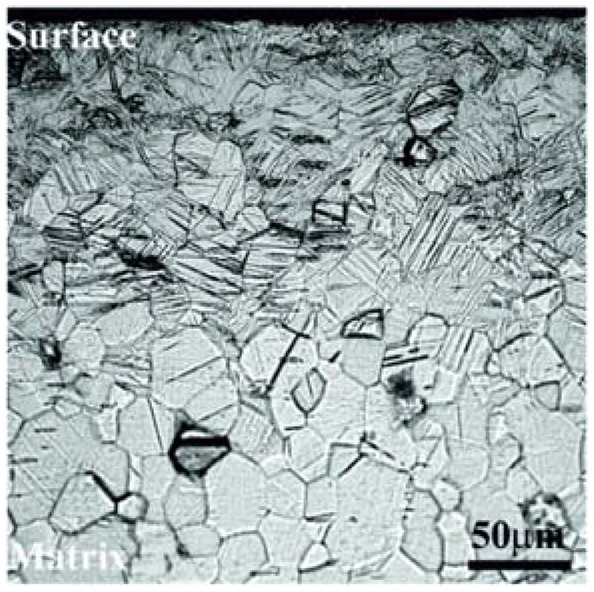
Optical micrograph of cross section of the SMATed-Ti. Reprinted from [55] with permission. Copyright (2013) IOP Publishing Ltd.

Another important factor for the formation of TiO_2_ nanotube arrays is the substrate microstructure. Pure titanium subjected to surface mechanical attrition treatment (SMAT-Ti) (Figure 7) and untreated were anodized in a glycol solution containing NH_4_F and small amounts of water [55]. Ti plates were annealed at 750 °C for 5 h to eliminate any residual deformation. After the annealing, they were ground to remove oxide and polished to a mirror finish. For each anodizing period the thickness of the TiO_2_ layer on SMATed-Ti was much thicker than that on unSMATed-Ti. The authors mentioned that nanocrystallized Ti is propitious to the growth of TiO_2_ nanotubes; grain boundaries and dislocations play the leading role in accelerating reaction rate and ion diffusion coefficient during anodization. The nanotube layers on unSMATed-Ti and SMATed-Ti were composed of amorphous TiO_2_. Nanocrystallization of Ti changed the surface morphologies and the phase components of TiO_2_ nanotubes.

Galstyan *et al.* studied how surface roughness of the substrates affects the morphology of TiO_2_ nanotubular arrays [46]. For the morphological analysis nanotubes were obtained on stiff and flexible substrates with different surface roughnesses. Initially metallic titanium films were deposited on flexible (Kapton® HN) and rough alumina substrates by means of RF (13.56 MHz) magnetron sputtering. Then the tubular arrays of TiO_2_ were prepared by electrochemical anodization in a glycerol- based electrolyte.

**Figure 8.**
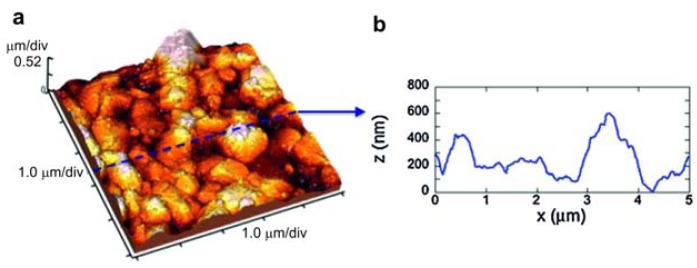
(**a**) 5 μm × 5 μm AFM image of the alumina substrate; (**b**) 5 μm line-scan profile measured from image (a). Reprinted from [46] with permission. Copyright (2013) Elsevier B.V.

**Figure 9.**
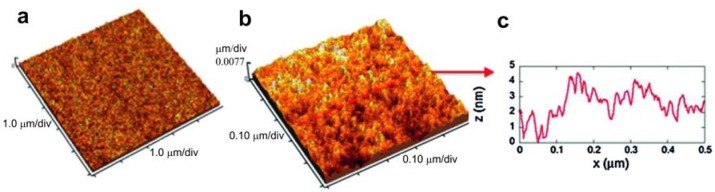
(**a**) 5 μm × 5 μm AFM image of the polymeric Kapton® HN substrate; (**b**) 0.5 μm × 0.5 μm AFM image of the polymeric Kapton® HN substrate; (**c**) 0.5 μm line-scan profile measured from image (b). Reprinted from [46] with permission. Copyright (2013) Elsevier B.V.

The surface analysis of the substrates showed a surface structure of stiff substrates composed by grains (alumina with the granular morphology) with measured sizes ranging from hundreds of nm to 1 μm (Figure 8), giving the substrate an RMS (root mean square) roughness of about 105 nm. Differently, the flexible polymeric substrate showed an almost flat surface (Figure 9) with an RMS roughness lower than 1 nm. Its morphology is better visualized in Figures 9b,c, where the 0.5 μm × 0.5 μm scan and a profile acquired along with it show the granularity of the polymer at the nanoscale, with grains having measured sizes of a few nm. SEM observations showed that well-aligned smooth tubes with a homogeneous distribution were obtained on both the substrates.

Nanotubes obtained on the granular surface of alumina substrates grew as a bunch of tubes on every grain. On the flexible substrate the same tube growth behavior did not take place due to the low roughness of the substrate, thus the surface roughness of the substrate also has an appreciable influence on the morphology of the resulting TiO_2_ tubular arrays.

**Figure 10.**
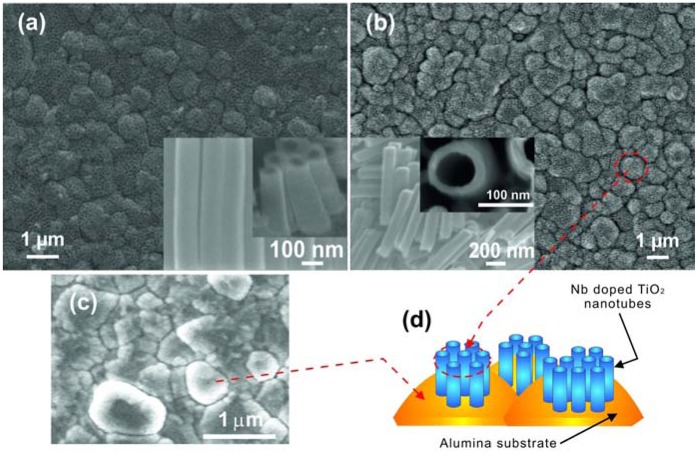
SEM images of (**a**) TiO_2_ and (**b**) Nb-doped TiO_2_ nanotubular arrays on alumina substrates obtained in 0.5 wt% NH_4_F and 2 mol L^−1^ H_2_O in glycerol at 90 V. (**c**) Surface of rough alumina substrate. (**d**) Schematic of the tubular structure on rough alumina substrate. Reprinted from [47] with permission. Copyright (2013) IOP Publishing Ltd.

In another work Galstyan *et al.* reported the properties of pure and doped TiO_2_ nanotubes prepared on a granular alumina surface with lateral dimensions of grains ranging between 200 and 1,200 nm [47]. Straight nanometric tubes were obtained on alumina decorating the grains of the substrate surface (Figure 10). The morphology of the tubular arrays formed on alumina [47] is different from those that are grown on Kapton® HN [46], but the distribution of tubes on both types of substrates is very homogenous.

Although the experimental results demonstrate that the electrochemical anodization method is a very convenient method for the modification of the surface structure of titania and allows obtaining highly ordered tubular arrays, the as-prepared structures are mainly amorphous. Usually the crystallization of the structures is carried out by post-growth annealing [38,47,54,56]. The amorphous to anatase or rutile phase transition is made possible by a variety of annealing regimes. The conversion from amorphous to crystalline anatase phase takes place at approximately 300 °C. The rutile phase appears at approximately 500 °C and becomes the dominant phase at 600 °C [56]. Improvement of structural and functional properties of titania nanostructures is an important issue for the fabrication of highly sensitive and selective sensing devices with a low working temperature. As we have mentioned above, the conductivity of chemical sensors changes due to the interaction between the sensing layer and gas molecules [3,4,9], therefore controlling the electronic, morphological and chemical properties of the sensing material, namely band-gap, Fermi level position, dispersion of catalyst, size of crystallites and their network connection is fundamental to enhance the sensitivity of chemoresistive devices [57–59]. Along with the development of geometrical parameters of TiO_2_ nanotubes recent research has concentrated on the enhancement of their structural and functional properties. Below recent advances in the fabrication of doped, mixed and modified TiO_2_ nanotubes that may improve the sensing properties of titania tubular structures are reported.

### 2.2. Fabrication of Doped, Mixed and Modified Titania Tubular Structures

**Figure 11.**
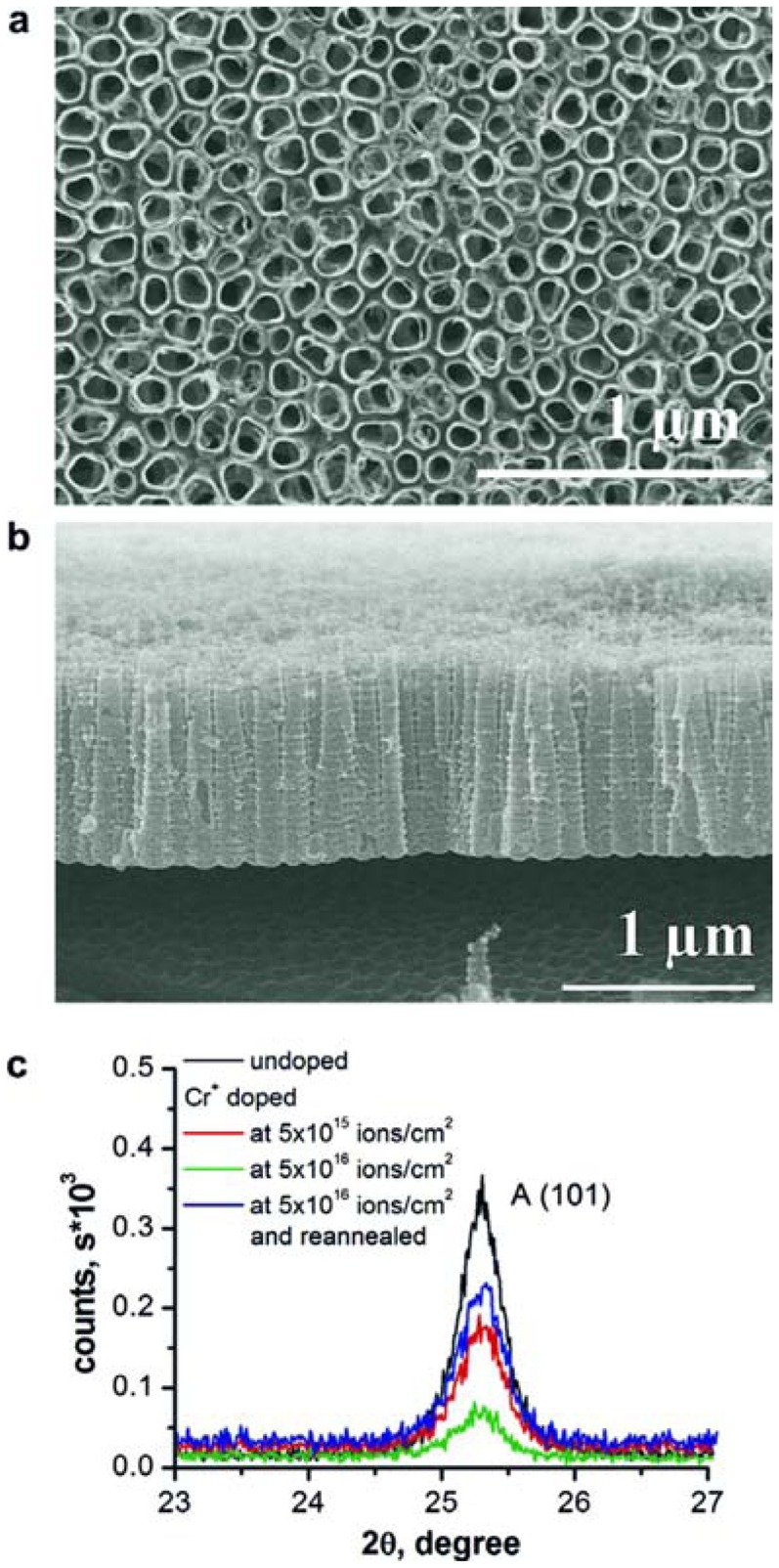
SEM top-view (**a**) and cross-section (**b**) of TiO_2_ nanotubes formed anodically in 1M (NaH_2_PO_4_) + 0.3wt%. HF electrolyte at 20 V for 2 h and subsequent annealed at 450 °C for 1 h, in air, and (**c**) the XRD results (anatase reflex – A) for the undoped samples, Cr^+^ ion implanted at nominal fluence 5 × 10^15^ cm^−2^, 5 × 10^16^ cm^−2^ and 5×10^16^ cm^−2^ with annealing. Reprinted from [60] with permission. Copyright (2013) Elsevier B.V.

During the last years the achievements in preparation of mixed and modified TiO_2_ nanotubes have been focused on the improvement of the structure properties for light harvesting, photocatalysis, bio-medical and sensing applications [8,60–73]. The nanotubes have been obtained mostly on metallic foil of titanium or its alloys [60,72–75]. Ghicov *et al.* reported doping of TiO_2_ nanotubes by an ion implantation method [60]. They prepared TiO_2_ nanotubes by anodization of metallic titanium and crystallized them by thermal annealing (Figure 11). Then they doped the crystallized structures with Cr using Cr^+^ ion implantation. Implantation was carried out in a 500 kV high voltage implanter using Cr^+^ ions at 60 keV accelerating energy and at two different nominal fluences of 1 × 10^15^ cm^−2^ and 1 × 10^16^ cm^−2^. After ion implantation the intensity of the anatase reflex decreases, indicating that the conversion of the crystalline form into an amorphous structure. The crystallization was recovered by re-heat treatment of the structure.

Ding *et al.* obtained Ti–Nb–O amorphous nanotubes on the top of titanium alloy surfaces [67]. The plate of Ti35Nb alloy (β-type Ti alloy with an elastic modulus of 68 GPa, the content of Nb is 35 wt%) was anodized in 1 M (NH_4_)_2_SO_4_ solution containing 0.5 wt% NH_4_F. Morphological analysis showed that the applied potential was crucial for the preparation of tubular arrays. At a lower anodization voltage of 10 V, only porous structures were prepared on the sample surface. When the anodization voltage was 15 V the tubes started to grow on the sample surface. The average inner diameter of the Ti–Nb–O nanotubes (60 nm) obtained at 15 V was also a few times bigger than the diameter of pores (20 nm) obtained at 10 V.

**Figure 12.**
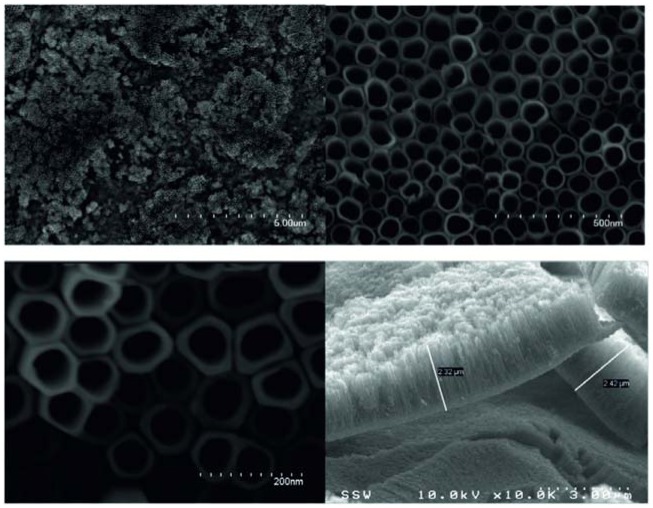
SEM images of Fe–C–N-codoped TiO_2_ nanotubes. Reprinted from [62] with permission. Copyright (2013) IOP Publishing Ltd.

Isimjan *et al.* doped titania tubes in the electrolyte during the growth [62]. Titanium foil was anodized in an ethylene glycol solution containing NH_4_F (0.38 wt%) and H_2_O (1.79 wt%) and placed in a well-insulated bath for 3 h at 30 V. Following the removal of the first anodized TiO_2_ nanotubular layer with adhesion tape a second anodization was performed for 3 h in glycol containing NH_4_F (0.38 wt%), H_2_O (1.79 wt%) and K_3_Fe(CN)_6_ (0.38 wt%) (Figure 12). Then the samples were annealed in air at 550 °C for 3 h. The structural analysis indicated that the tubes were doped with Fe, C and N. However, no appreciable signal related to Fe (281.5 eV) was observed by XPS, showing that the amount of Ti-bonded Fe was very low. EDX investigations clearly showed Fe signals in comparison to the un-doped TiO_2_ nanotubes. According to the composition of N (0.7%), the Fe composition was estimated at ∼0.1%.

However, for fabrication of chemical gas sensors the use of insulating substrates is an important condition, otherwise between the contacts for sensitivity measurements or between the contacts and the heater will cause current leakage. Very recently limited work has been done on the synthesis of doped TiO_2_ tubular arrays on insulating substrates. Galstyan *et al.* demonstrated the preparation of Nb-doped TiO_2_ nanotubes on alumina substrates [47]. The doped tubular arrays were obtained by anodization of metallic Nb-Ti films deposited on insulating alumina substrates (Figure 10). The deposition of metallic alloy was carried out by means of RF magnetron sputtering. The thickness of the metallic film was controlled by the sputtering regime. The anodization of the films was carried out at room temperature in a glycerol-based electrolyte. As-anodized tubular arrays were amorphous. The structural analysis indicated that after the thermal treatment, the Nb-doped TiO_2_ nanotubes were crystallized in the anatase structure, without any Nb oxide segregation. Introduction of Nb improved the conductivity of TiO_2_, which is an important feature for TiO_2_ as a chemical sensor candidate material. The method is also very convenient because the concentration of the dopant in the structure is possible to control by changing the composition of the target.

**Figure 13.**
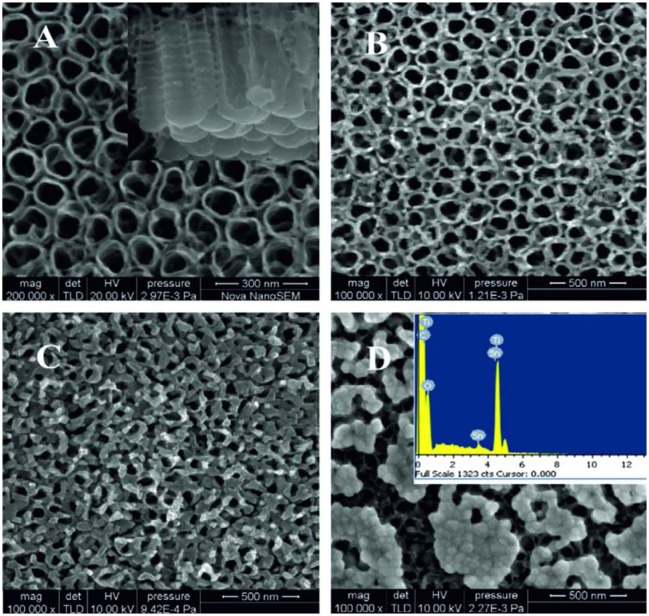
FESEM images of (**A**) C-TiO_2_NTs, (**B**) ST3, (**C**) ST5 and (**D**) ST7 (inset is the EDS spectrum). Reprinted from [61] with permission. Copyright (2013) Elsevier B.V.

Lai *et al.* also reported another interesting approach for the preparation of mixed TiO_2_ nanotubes in [76]. Tungsten trioxide (WO_3_) was incorporated throughout the walls of anodized TiO_2_ nanotubes by a wet impregnation method. Anodized TiO_2_ nanotube foil was dipped into an ammonium paratungstate (APT) aqueous solution with different molarities for 1 h. Subsequently, the samples were annealed at 400 °C, which decomposed the APT into tungsten trioxide (WO_3_). Meng *et al.* modified the TiO_2_ nanotubes in the autoclave [61]. Initially anodized TiO_2_ nanotubes were annealed in a CO atmosphere at 500 °C. Then the SnO_2_@C-TiO_2_ nanotubes were prepared by hydrothermal synthesis in a Teflon-lined stainless steel autoclave. The solution in the autoclave was 0.02 M SnCl·5H_2_O and 0.04 M NaOH in 50 mL deionized water. The samples were rinsed with distilled water several times in order to remove the residual reactant and then dried at 60 °C. When the reaction time was increased to 7 h (Figure 13), the SnO_2_ particles aggregated together on the nanotubes and the pores were blocked by the undesired nucleation and growth.

The abovementioned approaches for the synthesis of doped, mixed and modified titania nanotubes have been reported recently, and they still need to be improved. The fabrication of doped and mixed TiO_2_ nanostructures over a wide range of concentrations requires different approaches. Depending on the type and the concentration of dopant it is possible to change its functional properties [21,47,77–79], therefore it is very important to find the more suitable method for introduction of a specific mixture in titania nanotubes.

## 3. Gas Sensing Properties of Titania Nanotubes

### 3.1. Sensing Mechanism

Deviation of stoichiometry of high gap metal oxide is responsible of the semiconducting properties [80]: cation vacancies are acceptors, producing holes and negative charged vacancies, while shallow states made up of oxygen vacancies are double n-type donors, and the electrons on the adjacent cation are easily removed and donated to the conduction band [81].

The working temperature of metal oxide gas sensors ranges between 500 and 800 K in order to keep donor oxygen vacancies ionized but fixed. The gas sensing properties of metal oxides arise from the adsorption of molecules from the gas phase on reactive surface atoms. The first step of adsorption is physisorption, which is a slightly exothermic process characterized by high coverage at low temperature and a low coverage at high temperature. Physisorbed species can be chemisorbed (ionosorbed) when the adsorbate acts as a surface state capturing an electron or a hole [82]. In fact in the simple Charge Transfer Model (CTM) [83] the physi- and chemisorbed atoms and molecules are represented by surface localized states in the semiconductor energy gap, whose occupation statistic is given by the same Fermi-Dirac distribution, with physisorption corresponding to unoccupied and chemisorption to occupied states. The appearance of surface-localized acceptor states in n-type semiconductors induces charge transfer between bulk and surface in order to establish thermal equilibrium between the two. The charge transfer results in a non-neutral region (with a non-zero electric field) in the semiconductor bulk, usually referred to as the surface space charge region (SCR) [82,84]. The process of gas detection is related to the reactions between the species to be detected and ionosorbed surface oxygen [59]. Direct adsorption is also proposed for the gaseous species -like strongly electronegative NO_2_ whose effect is to decrease sensor conductance. An important ubiquitous species that ionosorbs over MOX surfaces is water; the chemisorption of water onto oxide from air can be very strong, forming a “hydroxylated surface” [82]. As for signal transduction, the easiest measurable physical quantity is the sensor conductance under DC conditions. The sensor response towards a target gas concentration is defined as the (relative) change of conductance (resistance in presence of NO_2_ or other oxidizing species). Starting from the sensor response it is possible to derive the sensor response curve, which is the representation of the steady state output as a function of the input concentration [85].

### 3.2. Fabrication of Sensing Devices

Concerning the integration of titania nanotubes into chemical sensing devices several approaches have been reported in the literature. These approaches may be divided firstly considering the eventual use of a substrate for the growth of nanotubes or the direct use of titanium foils. The majority of the literature data follow the second approach, which is the easiest method; nonetheless there are several drawbacks that have to be taken into account. First of all the reliability of the chemical sensing device is strongly dependent on the stability of the electrical contacts, and furthermore the contact preparation process must be as standard as possible in order to evaluate the performances of equivalent devices and not the ones of a single device.

Metal contacts are needed in order to measure electrical properties. The first sensing devices based on titania nanotubes were prepared using an anodized titanium foil and two platinum electrodes pressed onto the nanotubes [86]. Silver paste may be used also, but lack of reproducibility is an issue. Afterwards the anodization of titanium thin film was proposed, together with a direct integration in sensing devices [87] and metallic electrodes were evaporated onto the nanotubular array.

The use of films instead of a metallic foil allows for the direct integration of nanotubes on the transducer. The choice and the proper design of the transducer affect the response of the final device. Moreover a proper choice of bonding and packaging is needed to provide a final sensor device for practical applications.

**Figure 14.**
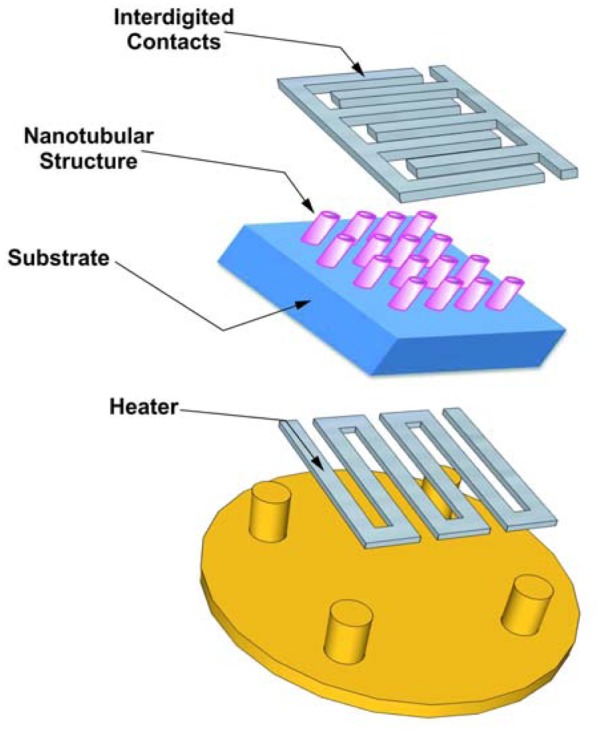
Design of a conductometric transducer.

Substrates for gas sensors are usually made of alumina or silicon. When the substrate is conductive an additional layer is required to guarantee insulation of the oxide layer. A heater is needed in order to maintain the layer at the appropriate temperature (up to hundreds of degrees centigrade). Alumina is one of the best substrates thanks to its availability with different roughnesses and crystalline properties. Figure 14 describes a design of the simplest transducer, a bulk substrate with the tubular structure and the electrical contacts on the front side of the substrate and a heater on the back side. In order to reach the desired operating temperature and to keep it constant on the titania nanotubes surface, a constant voltage may be applied to the heater with a feedback circuit to keep either resistance or power consumption constant by Joule heating.

Such transducers are prepared using thin or thick film technologies, the temperature uniformity is good, but unfortunately the power consumption is quite high, in the range 320–490 mW for 300–400 °C for a 2 × 2 mm substrate [88].

The choice of the metal for the electrical contacts is another key aspect, as it can affect the sensing properties of the final device. The contact has to provide as low a sheet resistance as possible in order to minimize the voltage drops between the connections, furthermore it should be ohmic. Platinum is of widespread use for metal oxides since it produces a good ohmic contact, it does not oxidize at high temperatures, it has a low diffusivity and it is resistive to corrosive gases. Gold on the other hand diffuses quickly and may introduce unintentional doping of titanium oxide nanotubes.

As the achievement of stable and reliable titania chemical sensors is important, a particular focus has to placed on the in-depth understanding of the detection mechanism and how the changes in material resistance influence the resistance variation of the entire device. The resistance modulation may be due to a change in the inter-grain potential barrier, but also in the metal semiconductor barrier at the electrical contacts. The latter may depend on the surrounding atmosphere and furthermore there may be possible chemical interactions due to the catalytic nature of the electrode (in general platinum, gold or silver), surface species may diffuse faster to the oxide interface where it can react (spill-over effect).

The most used measurement for chemical sensors is the two probe one that cannot separate the contributions of the active layer and contact resistance to the sensing properties. Changing the metal used for the electrical contacts and connections may influence the sensing performance, therefore great attention has to be paid to the overall device and not just to the sensing layer itself. In order to distinguish the contribution of the active layer four probe array analysis either in square or in collinear configuration may be used, but this increases the difficulties in the preparation of the device, and therefore is almost never proposed.

### 3.3. Sensing Performances

Starting from the first paper that pointed out the feasibility of using arrays of titania nanotubes as chemical sensors [89], effort has been made in the investigation of sensing performances of titania nanotubes. The majority of the works found in literature are based on nanotube arrays. In this case, a lot of nanotubes are contacted and contribute to the measured electrical and sensing properties. The uniformity of these arrays is generally good, the dispersion in the nanotubes diameter and spacing is low (20%) [90]. Making a single nanotube-based device has intrinsic technical difficulties for the manipulation of the structure and the preparation of reliable electrical contacts on one individual nanostructure with a fabrication process controlled at the nanoscale level. Chemical sensing performances will be classified on the base of the target species, and also on the base of key parameters such as gas carrier, humidity and operating temperatures (Table 3).

When dealing with chemical sensors there are several essential factors that we have to take into account and control carefully, first of all the gas carrier used for the dispersion of the target gas, then the relative humidity and moreover the operating temperature. In the majority of the applications of chemical sensors in real environments there is humid air as a background in which the gases may be detected, therefore the measurements should be made using humid air as gas carrier instead of nitrogen or synthetic air, otherwise the results obtained are not relevant for the preparation of chemical sensors. Oxygen and water vapor play a crucial role on the semiconducting surface and completely change the sensing performances. Sensors are heated in order to activate surface reactions with gaseous species [91].

Many authors did not follow this testing procedure, therefore the comparison of the results from different groups is quite difficult. A constant flux of gaseous species at ambient pressure should be preferred to an injection of desired amount of gas through a syringe in order to study the steady state gas-surface interaction.

The first report on titania nanotube sensing has been proposed in 2003 [89] using a furnace to control the nanotubes' temperature and hydrogen gas was dispersed in nitrogen (carrier gas). An extremely high variation in the electrical resistance was registered as 100–500 ppm of hydrogen was introduced in the test chamber at temperatures ranging between 300–400 °C. This variation in the nanotubes resistance was attributed to hydrogen chemisorption, and an increase in the variation with a decreasing pore diameter was measured.

Following this first report, other articles were published on the gas-surface interactions between titania nanotubes, prepared by anodization of metallic foil, and [12,92], oxygen and SO_2_ using nitrogen as gas carrier. The responses of amorphous titania nanotubes arrays towards 200 ppm of oxygen at 50–100 °C is reported in [93]. Response time was relatively long, but a complete recovery of the resistance in nitrogen was registered as the nitrogen flow was restored. Zhang *et al.* studied the interaction with 50 ppm of SO_2_ as a decomposition product of SF_6_ with anatase titania nanotubular arrays using silver glue for electrical connection and a furnace to operate from room temperature to 400 °C. The maximum value of the relative change in resistance is 0.8 approximately, relsatively low, but with a good reproducibility when UV light was used to help the desorption of SO_2_ molecules absorbed on the titania surface. Nitrogen dioxide and carbon monoxide interaction was reported in [94] using Ar as gas carrier, concentrations in the range 10–100 ppm and operating temperatures of 300–500 °C.

**Figure 15.**
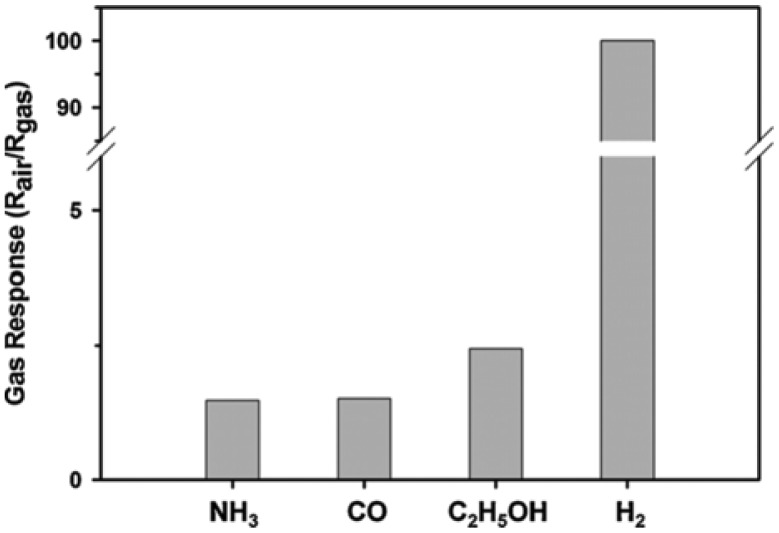
Magnitude of gas response of TiO_2_ nanotube sensor to 1,000 ppm of various gases at 100 °C. Reprinted from [95] with permission. Copyright (2013) Elsevier B.V.

The most interesting results for the integration of titania nanotubes into chemical sensors are the ones made for hydrogen [95], ethanol [39,72,87,95,96] ammonia [95,96] and carbon monoxide [39,95] in an air environment. Depending on the preparation technique selected, on the electrical contacts and the measurements technique, different performances have been reported. For example relatively high selectivity towards hydrogen at concentrations of 1,000 ppm with respect to ammonia, carbon monoxide and ethanol at an operating temperature of 100 °C have been reported in [95] (Figure 15). Furthermore the authors report also a good stability and repeatability, which are key aspects for the successful integration in sensing devices.

Chen and co-workers also reported good sensing performances towards hydrogen at room temperature using anodization from titanium foil, platinum or platinum/titanium contacts and the sensor was kept at room temperature [73]. The influence of the environment and electrical contacts was investigated showing a decrease in performance in the presence of oxygen and with Pt/Ti electrodes.

Reference [72] reported instead a relative variation in conductance of about 10^4^ to 1,000 ppm of ethanol at 250 °C; electrical contacts were made with silver glue and gold wires and the gas test measurements were made using a microsyringe to insert a concentration pulse in the environment close to the titania nanotubes array. Static measurements of ethanol and ammonia at room temperature on titania nanotubular arrays prepared by anodization of metallic foil and using a stainless steel crocodile as electrode for gas tests have been reported by Perrillo *et al.* [96].

Reference [39] reported sensing performances of titania nanotubes arrays prepared by anodization of titanium thin film integrated directly on alumina transducers and contacted with platinum interdigitated electrodes deposited by sputtering. The result showed good sensing performances toward carbon monoxide and ethanol at 200 °C using humid synthetic air as gas carrier.

Even if the majority of the research work published concerns nanotubes arrays, there is a publication on single nanotube based devices. A single nanotube was transferred onto a silicon wafer and was integrated into a field effect transistor [97], allowing the measurement of the electrical transport characteristics (electron mobility and electron concentration) of the individual nanotube, which are interesting parameters for conductometric chemical sensors. Furthermore its sensing performances towards humidity at room temperature were studied.

Actually the main drawback of using titania for chemical sensing is its high resistivity that does not allow an easy integration in chemical sensing systems. It requires expensive equipment for the signal readout. Research efforts have been made to increase the conductance of titania in the form of thin or thick films and also in forms of nanotubes.

**Figure 16.**
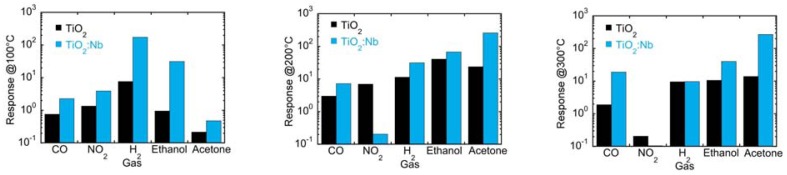
Response towards carbon monoxide, nitrogen dioxide, hydrogen, ethanol and acetone at 500 ppm, 10 ppm, 1,000 ppm, 500 ppm and 100 ppm, respectively and working temperatures of 100, 200 and 300 °C. Reprinted from [47] with permission. Copyright (2013) IOP Publishing Ltd.

Concerning chemical sensor preparation, investigations have been performed on the role of niobium doping on titania nanotubes conductance and chemical sensing performances [47,98]. Reference [47] reports the achievement of Nb-doped (0.14 at%) titania nanotubes by anodization of a Nb-Ti thin film. This concentration was enough to remarkably change the sensing performances (Figure 16) and the conductance, but the latter remained at values still not compatible with standard electronics. Further work of the same authors showed that a highly conducting titania nanotube array can be achieved by a room temperature synthesis of 5% wt Nb-containing titania nanotubular arrays. Results have shown that Nb does not segregate and effectively improves titania nanotube conductance to 10^−4^ S while maintaining good gas sensing performances.

## 4. Conclusions

Electrochemical anodization is by far the more performing technique for the synthesis, doping and modification of titania nanotubes. The surface morphology and the parameters of tubular arrays depend on the type, temperature and pH of the electrolyte, as well as the anodization time and the surface of the substrate. Good alignment and high ordering of tubes is straightforward to get reproducible gas sensing properties: thanks to their highly ordered and well-aligned structures, titania nanotubes have great potential applications in metal oxide gas sensors. Besides, doped and mixed oxide titania nanotubes with improved functional properties can be obtained by the anodization method. First of all doping improves the conductivity of TiO_2_, mainly in the high temperature range where the material is most sensitive to gaseous species. Given the high resistivity of pure TiO_2_, conductivity improvement is compulsory for low cost gas sensing. Furthermore introduction of dopants greatly enhances the gas sensing performance of TiO_2_ nanotubes.

## Figures and Tables

**Table 1.** Comparison of TiO_2_ nanotube preparation methods.

**References**	**Preparation Method**	**Growth Temperature (°C)**	**Crystal Structure**	**Possibility of Size Control**	**Template-Assisted Technique**	**Distribution over the Substrate**	**Starting Material**
[36–39,47]	Electro-chemical anodization	Room temperature	Amorphous	Yes	No	Well-ordered and vertically-aligned	Metallic Ti
[40,42]	ALD	90–400	Amorphous/ crystalline	Yes	Yes	Well-ordered and vertically-aligned	Precursor solution
[17,44,45]	Hydrothermal synthesis	300–450	Titanate, anatase	Yes	No	Chaotic distribution (powder)	TiO_2_ crystalline nanoparticulate powder, precursor solution

**Table 2.** Summary of the nanotube dimensions under different voltage and pH conditions and their maximum resistance variation to 1,000 ppm H_2_ at room temperature. The anodization time was 17 h for all samples. Reprinted from [8] with permission. Copyright (2013) IOP Publishing Ltd.

10 V (30 nm pore size)			
	Length	Log(*S*)	Log(*S*)
pH	(nm)	no Pd	with Pd
1.1	350	7.2	7.3
3.0	730	8.0	8.7
4.0	990	8.7	8.3
4.5	1400	8.0	8.3
5.0	2000	–	–

**Table 3.** List of the selected reference on chemical sensor based on titania nanotubes.

**Reference**	**Preparation**	**Contacts**	**Gas Carrier**	**Measurement**	**Gas**
[89]	Ti foil anodization	Pt	N_2_	Dynamic flow	H_2_, CO, NH_3_
[93]	Ti foil anodization	Pt	N_2_	Dynamic flow	O_2_
[12]	Ti foil anodization	Pt	N_2_	Dynamic flow	H_2_
[92]	Ti foil anodization	Au + Ag	N_2_	Dynamic flow	H_2_
[10]	Ti foil anodization	Ag	N_2_	Dynamic flow	SO_2_
[94]	Ti foil anodization	Pt	Ar	Dynamic flow	NO_2_, CO
[96]	Ti foil anodization	Stainless steel	Air	Static	Ethanol, NH_3_
[73]	Ti foil anodization	Pt	Ar	Static	H_2_
[72]	Ti foil anodization	Ag+Au	Air	Static	ethanol
[87]	Ti film on glass	Au	Air	Static	Volatile organic compounds
[95]	ALD	Pt	Air	Dynamic flow	H_2_, CO, ethanol, NH_3_
[98]	Ti35Nb foil anodization	Pt + Ag + Cu	Air, N_2_	Dynamic flow	H_2_
[39]	Ti film on alumina anodization	Pt	Air	Dynamic flow	CO, ethanol
[47]	Ti/Nb film on alumina anodization	Pt	Air	Dynamic flow	CO, NO_2_, H_2_, ethanol, acetone
